# Supplementary material for: Travel time to care does not affect survival for patients with colorectal cancer in northern Sweden: A data linkage study from the Risk North database
Source: PLoS One. 2020 Aug 5;15(8):e0236799. doi: 10.1371/journal.pone.0236799 (PMC7406033; doi:10.1371/journal.pone.0236799)
Supplement: S3 Fig — (DOCX) [file pone.0236799.s003.docx]

**S3 Fig. The patient´s education level vs. travel time to their nearest hospital.**
