# Supplementary material for: Travel time to care does not affect survival for patients with colorectal cancer in northern Sweden: A data linkage study from the Risk North database
Source: PLoS One. 2020 Aug 5;15(8):e0236799. doi: 10.1371/journal.pone.0236799 (PMC7406033; doi:10.1371/journal.pone.0236799)
Supplement: S1 Table — Hazard ratios of cause-specific survival in colorectal cancer for operated patients estimated in a multiple cox regression analysis; stratified by sex and age at diagnosis (10-year groups) and adjusted for educational level, cohabiting status, elective/emergency surgery and tumour stage. (DOCX) [file pone.0236799.s007.docx]

**S1 Table. Sensitivity analysis, results for analysing colorectal cancer as one entity.**

**Hazard ratios of cause-specific survival in colorectal cancer for operated patients estimated in a multiple cox regression analysis; stratified by sex and age at diagnosis (10-year groups) and adjusted for educational level, cohabiting status, elective/emergency surgery and tumour stage.**

|  | **Colorectal Cancer** | |
| --- | --- | --- |
|  | HR | 95% CI |
| **Travel time** | 0.999 | 0.997 -1.000 |
| **Education level** |  |  |
| Low (ref) | 1 (ref) |  |
| Medium | 0.93 | 0.78 – 1.11 |
| Higher | 0.90 | 0.71 – 1.13 |
| **Cohabitation status** |  |  |
| Living alone (ref) | 1(ref) |  |
| Not living alone | 0.78 | 0.66 – 0.91 |
| **Operation** |  |  |
| Elective (ref) | 1 (ref) |  |
| Emergency | 2.71 | 2.29 – 3.22 |
| **Tumour stage** |  |  |
| I (ref) | 1 (ref) |  |
| II | 1.83 | 1.21 – 2.79 |
| III | 5.22 | 3.53 – 7.72 |
| IV | 21.1 | 13.19 – 31.30 |
