# Supplementary material for: Travel time to care does not affect survival for patients with colorectal cancer in northern Sweden: A data linkage study from the Risk North database
Source: PLoS One. 2020 Aug 5;15(8):e0236799. doi: 10.1371/journal.pone.0236799 (PMC7406033; doi:10.1371/journal.pone.0236799)
Supplement: S2 Table — Hazard ratios of all-cause survival for operated patients estimated in a multiple cox regression analysis; stratified by sex and age at diagnosis (10-year groups) and adjusted for educational level, cohabiting status, elective/emergency surgery and tumour stage. (DOCX) [file pone.0236799.s008.docx]

**S2 Table. Sensitivity analysis, results for analysing all-cause survival.**

**Hazard ratios of all-cause survival for operated patients estimated in a multiple cox regression analysis; stratified by sex and age at diagnosis (10-year groups) and adjusted for educational level, cohabiting status, elective/emergency surgery and tumour stage.**

|  | **Colon Cancer** | | **Rectal Cancer** | |
| --- | --- | --- | --- | --- |
|  | HR | 95% CI | HR | 95% CI |
| **Travel time** | 1.000 | 0.998 -1.002 | 0.998 | 0.994 – 1.002 |
| **Education level** |  |  |  |  |
| Low (ref) | 1 (ref) |  | 1 |  |
| Medium | 0.93 | 0.79 – 1.10 | 0.81 | 0.60 – 1.10 |
| Higher | 0.86 | 0.69 – 1.07 | 0.99 | 0.67 – 1.46 |
| **Cohabitation status** |  |  |  |  |
| Living alone (ref) | 1(ref) |  | 1 |  |
| Not living alone | 0.75 | 0.65 – 0.88 | 0.69 | 0.52 – 0.90 |
| **Operation** |  |  |  |  |
| Elective (ref) | 1 (ref) |  | 1 |  |
| Emergency | 2.32 | 1.99 – 2.71 | 5.87 | 3.14 – 11.0 |
| **Tumour stage** |  |  |  |  |
| I (ref) | 1 (ref) |  | 1 |  |
| II | 1.14 | 0.85 – 1.55 | 1.86 | 1.18 – 2.92 |
| III | 2.39 | 1.80 – 3.17 | 2.69 | 1.17 – 4.17 |
| IV | 8.11 | 6.06 – 10.9 | 10.2 | 6.29 – 16.5 |
