# Supplementary material for: Travel time to care does not affect survival for patients with colorectal cancer in northern Sweden: A data linkage study from the Risk North database
Source: PLoS One. 2020 Aug 5;15(8):e0236799. doi: 10.1371/journal.pone.0236799 (PMC7406033; doi:10.1371/journal.pone.0236799)
Supplement: S3 Table — Hazard ratios of cause specific survival for operated patients estimated in a multiple cox regression analysis; stratified by sex and age at diagnosis (10-year groups) and adjusted for educational level and cohabiting status. (DOCX) [file pone.0236799.s009.docx]

**S3 Table. Sensitivity analysis, results for analysing cause-specific survival without adjusting for tumour stage and emergency operations.**

**Hazard ratios of cause specific survival for operated patients estimated in a multiple cox regression analysis; stratified by sex and age at diagnosis (10-year groups) and adjusted for educational level and cohabiting status.**

|  | **Colon Cancer** | | **Rectal Cancer** | |
| --- | --- | --- | --- | --- |
|  | HR | 95% CI | HR | 95% CI |
| **Travel time** | 1.000 | 0.998 -1.002 | 0.998 | 0.995 – 1.001 |
| **Education level** |  |  |  |  |
| Low (ref) | 1 (ref) |  | 1 |  |
| Medium | 0.92 | 0.78 – 1.00 | 0.90 | 0.71 – 1.15 |
| Higher | 0.96 | 0.78 – 1.19 | 0.96 | 0.70 – 1.32 |
| **Cohabitation status** |  |  |  |  |
| Living alone (ref) | 1(ref) |  | 1 |  |
| Not living alone | 0.85 | 0.73 – 0.99 | 0.78 | 0.63 – 0.97 |
