# Supplementary material for: Travel time to care does not affect survival for patients with colorectal cancer in northern Sweden: A data linkage study from the Risk North database
Source: PLoS One. 2020 Aug 5;15(8):e0236799. doi: 10.1371/journal.pone.0236799 (PMC7406033; doi:10.1371/journal.pone.0236799)
Supplement: S4 Table — Hazard ratios of cause-specific survival for operated patients estimated in a multiple cox regression analysis; stratified by sex and age at diagnosis (10-year groups) and adjusted for educational level, cohabiting status, elective/emergency surgery and tumour stage. (DOCX) [file pone.0236799.s010.docx]

**S4 Table. Sensitivity analysis, results for analysing travel time to the operating hospital.**

**Hazard ratios of cause-specific survival for operated patients estimated in a multiple cox regression analysis; stratified by sex and age at diagnosis (10-year groups) and adjusted for educational level, cohabiting status, elective/emergency surgery and tumour stage.**

|  | **Colon Cancer** | | **Rectal Cancer** | |
| --- | --- | --- | --- | --- |
|  | HR | 95% CI | HR | 95% CI |
| **Travel time** | 0.999 | 0.997 -1.000 | 0.998 | 1.000 – 1.001 |
| **Education level** |  |  |  |  |
| Low (ref) | 1 (ref) |  | 1 |  |
| Medium | 0.94 | 0.77 – 1.15 | 0.87 | 0.61 – 1.25 |
| Higher | 0.87 | 0.66 – 1.13 | 1.04 | 0.66 – 1.66 |
| **Cohabitation status** |  |  |  |  |
| Living alone (ref) | 1(ref) |  | 1 |  |
| Not living alone | 0.77 | 0.63 – 0.92 | 0.79 | 0.57 – 1.09 |
| **Operation** |  |  |  |  |
| Elective (ref) | 1 (ref) |  | 1 |  |
| Emergency | 2.67 | 2.24 – 3.25 | 5.4 | 2.61 – 11.0 |
| **Tumour stage** |  |  |  |  |
| I (ref) | 1 (ref) |  | 1 |  |
| II | 1.60 | 0.91 – 2.81 | 2.87 | 1.50 – 5.47 |
| III | 6.17 | 3.64 – 10.4 | 4.05 | 2.17 – 7.56 |
| IV | 22.8 | 13.4 – 38.7 | 22.0 | 11.4 – 42.2 |
