# Supplementary material for: Travel time to care does not affect survival for patients with colorectal cancer in northern Sweden: A data linkage study from the Risk North database
Source: PLoS One. 2020 Aug 5;15(8):e0236799. doi: 10.1371/journal.pone.0236799 (PMC7406033; doi:10.1371/journal.pone.0236799)
Supplement: S6 Table — Hazard ratios of cause-specific survival for operated patients estimated in a multiple cox regression analysis; stratified by sex and age at diagnosis (10-year groups) and adjusted for educational level, cohabiting status, elective/emergency surgery and tumour stage. (DOCX) [file pone.0236799.s012.docx]

**S6 Table. Additional setting of the multivariable regression survival analysis, travel time handled as a categorical variable; survival for patients with travel time < 1 h compared to patients traveling > 1 h.**

**Hazard ratios of cause-specific survival for operated patients estimated in a multiple cox regression analysis; stratified by sex and age at diagnosis (10-year groups) and adjusted for educational level, cohabiting status, elective/emergency surgery and tumour stage.**

|  | **Colon Cancer** | | **Rectal Cancer** | |
| --- | --- | --- | --- | --- |
|  | HR | 95% CI | HR | 95% CI |
| **Travel time**  0-59 minutes (ref)  1 h or more | 1  0.92 | 0.71 -1.19 | 1  0.84 | 0.52 – 1.36 |
| **Education level** |  |  |  |  |
| Low (ref) | 1 (ref) |  | 1 |  |
| Medium | 0.96 | 0.78 – 1.17 | 0.86 | 0.60 – 1.23 |
| Higher | 0.87 | 0.67 – 1.14 | 1.06 | 0.67 – 1.68 |
| **Cohabitation status** |  |  |  |  |
| Living alone (ref) | 1(ref) |  | 1 |  |
| Not living alone | 0.77 | 0.64 – 0.93 | 0.78 | 0.57 – 1.07 |
| **Operation** |  |  |  |  |
| Elective (ref) | 1 (ref) |  | 1 |  |
| Emergency | 2.65 | 2.21 – 3.19 | 5.50 | 2.68 – 11.3 |
| **Tumour stage** |  |  |  |  |
| I (ref) | 1 (ref) |  | 1 |  |
| II | 1.66 | 0.94 – 2.91 | 2.77 | 1.45 – 5.30 |
| III | 6.15 | 3.63 – 10.4 | 4.03 | 2.16 – 7.51 |
| IV | 22.9 | 13.4 – 38.8 | 20.7 | 10.8 – 39.7 |
