# Supplementary material for: Travel time to care does not affect survival for patients with colorectal cancer in northern Sweden: A data linkage study from the Risk North database
Source: PLoS One. 2020 Aug 5;15(8):e0236799. doi: 10.1371/journal.pone.0236799 (PMC7406033; doi:10.1371/journal.pone.0236799)
Supplement: S1 File — (DOCX) [file pone.0236799.s013.docx]

# Regression analyses

## Cox main model – travel time as continuous variable, Colon cancer

Call:

coxph(formula = SurvObj_crc ~ FromBreak + as.factor(utbildning) +

factor(AntalPersoner %in% 1, levels = c(T, F)) + factor(akutelektiv) +

as.factor(stadium) + strata(as.factor(careg_sex), cut(careg_alder,

breaks = c(0, 10, 20, 30, 40, 50, 60, 70, 80, 90, Inf))),

data = tabdata1[tabdata1$A2_tumlok_Beskrivning %in% "Colon",

])

n= 2010, number of events= 509

(453 observations deleted due to missingness)

coef exp(coef) se(coef) z Pr(>|z|)

FromBreak -0.0007146 0.9992857 0.0013152 -0.543 0.58691

as.factor(utbildning)34Gymnasial -0.0423751 0.9585101 0.1027559 -0.412 0.68006

as.factor(utbildning)567Eftergymnasial -0.1359826 0.8728578 0.1358298 -1.001 0.31677

factor(AntalPersoner %in% 1, levels = c(T, F))FALSE -0.2613393 0.7700196 0.0951717 -2.746 0.00603 **

factor(akutelektiv)2_akut 0.9790037 2.6618030 0.0945251 10.357 < 2e-16 ***

as.factor(stadium)2 0.5050376 1.6570477 0.2880136 1.754 0.07951 .

as.factor(stadium)3 1.8170757 6.1538362 0.2686826 6.763 1.35e-11 ***

as.factor(stadium)4 3.1310138 22.8971799 0.2705861 11.571 < 2e-16 ***

---

Signif. codes: 0 ‘***’ 0.001 ‘**’ 0.01 ‘*’ 0.05 ‘.’ 0.1 ‘ ’ 1

exp(coef) exp(-coef) lower .95 upper .95

FromBreak 0.9993 1.00071 0.9967 1.0019

as.factor(utbildning)34Gymnasial 0.9585 1.04329 0.7837 1.1724

as.factor(utbildning)567Eftergymnasial 0.8729 1.14566 0.6688 1.1391

factor(AntalPersoner %in% 1, levels = c(T, F))FALSE 0.7700 1.29867 0.6390 0.9279

factor(akutelektiv)2_akut 2.6618 0.37569 2.2116 3.2036

as.factor(stadium)2 1.6570 0.60348 0.9423 2.9140

as.factor(stadium)3 6.1538 0.16250 3.6345 10.4196

as.factor(stadium)4 22.8972 0.04367 13.4728 38.9139

Concordance= 0.805 (se = 0.011 )

Likelihood ratio test= 634.8 on 8 df, p=<2e-16

Wald test = 577.8 on 8 df, p=<2e-16

Score (logrank) test = 835.5 on 8 df, p=<2e-16

## Additional cox model – travel time as categorical variable, Colon cancer

Call:

coxph(formula = SurvObj_crc ~ factor(restid, levels = c("< 1h",

">= 1h")) + as.factor(utbildning) + factor(AntalPersoner %in%

1, levels = c(T, F)) + factor(akutelektiv) + as.factor(stadium) +

strata(as.factor(careg_sex), cut(careg_alder, breaks = c(0,

10, 20, 30, 40, 50, 60, 70, 80, 90, Inf))), data = tabdata1[tabdata1$A2_tumlok_Beskrivning %in%

"Colon", ])

n= 2010, number of events= 509

(453 observations deleted due to missingness)

coef exp(coef) se(coef) z Pr(>|z|)

factor(restid, levels = c("< 1h", ">= 1h"))>= 1h -0.08559 0.91797 0.13159 -0.650 0.51542

as.factor(utbildning)34Gymnasial -0.04261 0.95829 0.10269 -0.415 0.67820

as.factor(utbildning)567Eftergymnasial -0.13550 0.87328 0.13567 -0.999 0.31793

factor(AntalPersoner %in% 1, levels = c(T, F))FALSE -0.26384 0.76809 0.09531 -2.768 0.00564 **

factor(akutelektiv)2_akut 0.97622 2.65439 0.09451 10.329 < 2e-16 ***

as.factor(stadium)2 0.50430 1.65583 0.28799 1.751 0.07992 .

as.factor(stadium)3 1.81588 6.14646 0.26865 6.759 1.39e-11 ***

as.factor(stadium)4 3.12898 22.85056 0.27049 11.568 < 2e-16 ***

---

Signif. codes: 0 ‘***’ 0.001 ‘**’ 0.01 ‘*’ 0.05 ‘.’ 0.1 ‘ ’ 1

exp(coef) exp(-coef) lower .95 upper .95

factor(restid, levels = c("< 1h", ">= 1h"))>= 1h 0.9180 1.08936 0.7093 1.1881

as.factor(utbildning)34Gymnasial 0.9583 1.04353 0.7836 1.1719

as.factor(utbildning)567Eftergymnasial 0.8733 1.14511 0.6694 1.1393

factor(AntalPersoner %in% 1, levels = c(T, F))FALSE 0.7681 1.30192 0.6372 0.9259

factor(akutelektiv)2_akut 2.6544 0.37673 2.2055 3.1946

as.factor(stadium)2 1.6558 0.60393 0.9416 2.9117

as.factor(stadium)3 6.1465 0.16270 3.6304 10.4063

as.factor(stadium)4 22.8506 0.04376 13.4480 38.8272

Concordance= 0.805 (se = 0.011 )

Likelihood ratio test= 634.9 on 8 df, p=<2e-16

Wald test = 578 on 8 df, p=<2e-16

Score (logrank) test = 835.9 on 8 df, p=<2e-16

## Cox main model – travel time as continuous variable , Rectal cancer

Call:

coxph(formula = SurvObj_crc ~ FromBreak + as.factor(utbildning) +

factor(AntalPersoner %in% 1, levels = c(T, F)) + factor(akutelektiv) +

as.factor(stadium) + strata(as.factor(careg_sex), cut(careg_alder,

breaks = c(0, 10, 20, 30, 40, 50, 60, 70, 80, 90, Inf))),

data = tabdata1[tabdata1$A2_tumlok_Beskrivning %in% "Rektum",

])

n= 927, number of events= 179

(326 observations deleted due to missingness)

coef exp(coef) se(coef) z Pr(>|z|)

FromBreak -0.003177 0.996828 0.002639 -1.204 0.22858

as.factor(utbildning)34Gymnasial -0.148817 0.861727 0.183183 -0.812 0.41657

as.factor(utbildning)567Eftergymnasial 0.045956 1.047028 0.235816 0.195 0.84549

factor(AntalPersoner %in% 1, levels = c(T, F))FALSE -0.257306 0.773132 0.163239 -1.576 0.11497

factor(akutelektiv)2_akut 1.707071 5.512792 0.366803 4.654 3.26e-06 ***

as.factor(stadium)2 1.013698 2.755773 0.329681 3.075 0.00211 **

as.factor(stadium)3 1.380775 3.977984 0.318064 4.341 1.42e-05 ***

as.factor(stadium)4 3.031648 20.731375 0.331786 9.137 < 2e-16 ***

---

Signif. codes: 0 ‘***’ 0.001 ‘**’ 0.01 ‘*’ 0.05 ‘.’ 0.1 ‘ ’ 1

exp(coef) exp(-coef) lower .95 upper .95

FromBreak 0.9968 1.00318 0.9917 1.002

as.factor(utbildning)34Gymnasial 0.8617 1.16046 0.6018 1.234

as.factor(utbildning)567Eftergymnasial 1.0470 0.95508 0.6595 1.662

factor(AntalPersoner %in% 1, levels = c(T, F))FALSE 0.7731 1.29344 0.5614 1.065

factor(akutelektiv)2_akut 5.5128 0.18140 2.6863 11.313

as.factor(stadium)2 2.7558 0.36287 1.4442 5.259

as.factor(stadium)3 3.9780 0.25138 2.1327 7.420

as.factor(stadium)4 20.7314 0.04824 10.8196 39.723

Concordance= 0.763 (se = 0.022 )

Likelihood ratio test= 167 on 8 df, p=<2e-16

Wald test = 184.7 on 8 df, p=<2e-16

Score (logrank) test = 300 on 8 df, p=<2e-16

## Additional cox model – travel time as categorical variable, Rectal cancer

Call:

coxph(formula = SurvObj_crc ~ factor(restid, levels = c("< 1h",

">= 1h")) + as.factor(utbildning) + factor(AntalPersoner %in%

1, levels = c(T, F)) + factor(akutelektiv) + as.factor(stadium) +

strata(as.factor(careg_sex), cut(careg_alder, breaks = c(0,

10, 20, 30, 40, 50, 60, 70, 80, 90, Inf))), data = tabdata1[tabdata1$A2_tumlok_Beskrivning %in%

"Rektum", ])

n= 927, number of events= 179

(326 observations deleted due to missingness)

coef exp(coef) se(coef) z Pr(>|z|)

factor(restid, levels = c("< 1h", ">= 1h"))>= 1h -0.17030 0.84341 0.24394 -0.698 0.48510

as.factor(utbildning)34Gymnasial -0.14955 0.86110 0.18359 -0.815 0.41531

as.factor(utbildning)567Eftergymnasial 0.05689 1.05854 0.23588 0.241 0.80941

factor(AntalPersoner %in% 1, levels = c(T, F))FALSE -0.25034 0.77854 0.16316 -1.534 0.12495

factor(akutelektiv)2_akut 1.70439 5.49801 0.36662 4.649 3.34e-06 ***

as.factor(stadium)2 1.02026 2.77391 0.32996 3.092 0.00199 **

as.factor(stadium)3 1.39371 4.02975 0.31789 4.384 1.16e-05 ***

as.factor(stadium)4 3.03097 20.71736 0.33178 9.136 < 2e-16 ***

---

Signif. codes: 0 ‘***’ 0.001 ‘**’ 0.01 ‘*’ 0.05 ‘.’ 0.1 ‘ ’ 1

exp(coef) exp(-coef) lower .95 upper .95

factor(restid, levels = c("< 1h", ">= 1h"))>= 1h 0.8434 1.18566 0.5229 1.360

as.factor(utbildning)34Gymnasial 0.8611 1.16131 0.6009 1.234

as.factor(utbildning)567Eftergymnasial 1.0585 0.94470 0.6667 1.681

factor(AntalPersoner %in% 1, levels = c(T, F))FALSE 0.7785 1.28446 0.5655 1.072

factor(akutelektiv)2_akut 5.4980 0.18188 2.6800 11.279

as.factor(stadium)2 2.7739 0.36050 1.4529 5.296

as.factor(stadium)3 4.0298 0.24815 2.1612 7.514

as.factor(stadium)4 20.7174 0.04827 10.8125 39.696

Concordance= 0.761 (se = 0.022 )

Likelihood ratio test= 166 on 8 df, p=<2e-16

Wald test = 184.5 on 8 df, p=<2e-16

Score (logrank) test = 299.3 on 8 df, p=<2e-16
