# Supplementary material for: Travel time to care does not affect survival for patients with colorectal cancer in northern Sweden: A data linkage study from the Risk North database
Source: PLoS One. 2020 Aug 5;15(8):e0236799. doi: 10.1371/journal.pone.0236799 (PMC7406033; doi:10.1371/journal.pone.0236799)
Supplement: Attachment — Submitted filename: Respons to Reviewers.docx [file pone.0236799.s015.docx]

Dear Editor!

Thank you for your further comments to the manuscript. We have adjusted the manuscript according to your suggestions. Hopefully the manuscript is now acceptable!

Our responds are given point-by-point below and highlighted in the manuscript.

Best Wishes, Olle Sjöström, Corresponding author.

One reviewer's comments;

They do not offer a clear suggestion as to why this might be apart from indicating the subsidised travel for patients' attendance at hospital. My question is, do the Northern Swedish patients have good outcomes because they have a culture which enables them to compensate for their isolation? In this respect, is there anything to be learned from the study by Kradval (now ref 26) comparing the north-south geography of Norway with that of Sweden?

We agree with Dr S. Michael Crawford (Reviewer #1) that the reasons to the good outcome in Northern Sweden might be found in protective factors not related the health care system. Differences on a health care system level with possible effects on the association between survival and distance to care are however easier to discuss since references describing different health care systems are more available and distinct. Psychosocial factor would be more speculative to discuss as we do not have any data to support it.

In the study by Kravdal et al the authors speculate that individuals living in Northern Norway - due to psychological or social factors are more used to travel far in their everyday life - thus reducing travel distance as a barrier to care. They also discuss the possibility of a healthier lifestyle among the population outside the city centres - resulting in a more favourable prognosis. Neither our study nor the study by Kravdal et al have however any results to support these by all means much plausible hypothesises.

But in order to broaden the discussion to factors outside the health care system we have added the following in the Discussion:

*“ Finally, there might be other factors - not related to the health care system - which explains the good outcome in Northern Sweden. As suggested in a Norwegian study, individuals living in rural areas might be more used to travel far in their everyday life - thus reducing travel distance as a barrier to care.^28^ ”*

A few minor changes are required.

1) please provide a citation for  "The Northern Healthcare Region has approximately 900 000 inhabitants
living in an area of 224 000 km2 , resulting in a population density of only 4 pop./km2(U.K. 274 pop./km2 ,France 123 pop./km2)

Please see the revised part of the Introduction and new references 14 and 15.

2) please specify whether authors used a two-sided test when presenting a significance level of .05.

We have changed the paragraph “*To test for proposed associations, we used two-tailed between-subject Student’s t-test for continuous parametric variables and Spearman’s test for non-parametric ranked variables (α=0.05)*” to ”*To test for proposed associations, we used two-sided between-subject Student’s t-test for continuous parametric variables and Spearman’s test for non-parametric ranked variables (α=0.05)”*

3) In figure 2, please add tick marks on the X axis. For example, where is 110 min (n=110 (3%))? It is hard to figure it out exactly.  It is all the same color beyond 80-90 min.  Please also write "(minutes)" on the X axis label :Travel time (minutes)

We have revised Figure 2 according to your suggestions, which made the histogram much easier to understand!

4) In table 2, what statistical method was used to obtain p values for either continuous or categorical variable?

Please see the revised version of Table 2, footnotes indicating statistical method has been added.

Additional Editor Comments (if provided):

There are minor comments as follows.

Please specify statistical tests that authors used for new Table 2.

Please see 4) above!

When authors discuss health care system in Sweden compared to other countries, please provide information on some statistics on health care delivery in Sweden to get clearer idea (e.g. population density, hospital facilities per population, fewer hospitals for surgical treatment for rectal cancer-> did authors have some numbers?).

Please see the revised section of the discussion:

*”All 13 hospitals in the Northern Health Care Region are publicly owned and all have facilities to diagnose colorectal cancer. Each local hospital offers service to the population in its catchment area. The population, which is served per hospital, varies from approximately 25000 to 160000.^14^ Surgical treatment, especially for rectal cancer, has however been centralised to fewer hospitals in the Region during the study period. By the end of the study period, rectal cancer surgery was performed at only five hospitals. ”*

[Note: HTML markup is below. Please do not edit.]

Reviewers' comments:

Reviewer's Responses to Questions

**Comments to the Author**

1. If the authors have adequately addressed your comments raised in a previous round of review and you feel that this manuscript is now acceptable for publication, you may indicate that here to bypass the “Comments to the Author” section, enter your conflict of interest statement in the “Confidential to Editor” section, and submit your "Accept" recommendation.

Reviewer #1: (No Response)

Reviewer #2: All comments have been addressed

2. Is the manuscript technically sound, and do the data support the conclusions?

The manuscript must describe a technically sound piece of scientific research with data that supports the conclusions. Experiments must have been conducted rigorously, with appropriate controls, replication, and sample sizes. The conclusions must be drawn appropriately based on the data presented. 

Reviewer #1: Yes

Reviewer #2: Yes

3. Has the statistical analysis been performed appropriately and rigorously? 

Reviewer #1: I Don't Know

Reviewer #2: Yes

4. Have the authors made all data underlying the findings in their manuscript fully available?

The [PLOS Data policy](https://eur02.safelinks.protection.outlook.com/?url=http%3A%2F%2Fwww.plosone.org%2Fstatic%2Fpolicies.action%23sharing&data=02%7C01%7Colle.sjostrom%40regionjh.se%7C8b01dd9ea74b4f58e02d08d816cd729d%7Cd3b4cf3aca774a02aefaf4398591468f%7C0%7C0%7C637284418935175631&sdata=%2BqFWVP3%2BVwzJECUX80OlmFqreeTJqean%2FVPdW4joi8M%3D&reserved=0) requires authors to make all data underlying the findings described in their manuscript fully available without restriction, with rare exception (please refer to the Data Availability Statement in the manuscript PDF file). The data should be provided as part of the manuscript or its supporting information, or deposited to a public repository. For example, in addition to summary statistics, the data points behind means, medians and variance measures should be available. If there are restrictions on publicly sharing data—e.g. participant privacy or use of data from a third party—those must be specified.

Reviewer #1: Yes

Reviewer #2: Yes

5. Is the manuscript presented in an intelligible fashion and written in standard English?

PLOS ONE does not copyedit accepted manuscripts, so the language in submitted articles must be clear, correct, and unambiguous. Any typographical or grammatical errors should be corrected at revision, so please note any specific errors here.

Reviewer #1: Yes

Reviewer #2: Yes

6. Review Comments to the Author

Please use the space provided to explain your answers to the questions above. You may also include additional comments for the author, including concerns about dual publication, research ethics, or publication ethics. (Please upload your review as an attachment if it exceeds 20,000 characters)

Reviewer #1: This is a much clearer presentation of the data and the points have all been addressed with the exception of one, where the authors did not understand my point! To express it differently, the authors show the lack of distance decay in outcomes from colorectal cancer and also point out that these findings differ from the consensus in other literature. They do not offer a clear suggestion as to why this might be apart from indicating the subsidised travel for patients' attendance at hospital. My question is, do the Northern Swedish patients have good outcomes because they have a culture which enables them to compensate for their isolation? In this respect, is there anything to be learned from the study by Kradval (now ref 26) comparing the north-south geography of Norway with that of Sweden?

Please see above!

Reviewer #2: The authors have addressed the concerns of the reviewers well. I appreciated the additional context about the population evaluated in this paper.

7. PLOS authors have the option to publish the peer review history of their article ([what does this mean?](https://eur02.safelinks.protection.outlook.com/?url=https%3A%2F%2Fjournals.plos.org%2Fplosone%2Fs%2Feditorial-and-peer-review-process%23loc-peer-review-history&data=02%7C01%7Colle.sjostrom%40regionjh.se%7C8b01dd9ea74b4f58e02d08d816cd729d%7Cd3b4cf3aca774a02aefaf4398591468f%7C0%7C0%7C637284418935185622&sdata=RW9g9huaNBLtxS%2Fhric8a4GvUFOm9R5H4LC%2FIyHlH6g%3D&reserved=0)). If published, this will include your full peer review and any attached files.


If you choose “no”, your identity will remain anonymous but your review may still be made public.


**Do you want your identity to be public for this peer review?** For information about this choice, including consent withdrawal, please see our [Privacy Policy](https://eur02.safelinks.protection.outlook.com/?url=https%3A%2F%2Fwww.plos.org%2Fprivacy-policy&data=02%7C01%7Colle.sjostrom%40regionjh.se%7C8b01dd9ea74b4f58e02d08d816cd729d%7Cd3b4cf3aca774a02aefaf4398591468f%7C0%7C0%7C637284418935195616&sdata=4tSJ%2FwV%2BSUAEQxzvD%2BkOcUFObEMxaA%2FWe0uCNe6TIgw%3D&reserved=0).

Reviewer #1: Yes: Dr S. Michael Crawford

Reviewer #2: No
